# Genomic insights into *Kocuria*: taxonomic revision and identification of five IAA-producing extremophiles

**DOI:** 10.3389/fmicb.2025.1547983

**Published:** 2025-05-27

**Authors:** Cong-Jian Li, Zhu-Ming Jiang, Xiao-Yang Zhi, Hua-Hong Chen, Li-Yan Yu, Guang-Fu Li, Yu-Qin Zhang

**Affiliations:** ^1^Institute of Medicinal Biotechnology, Chinese Academy of Medical Sciences & Peking Union Medical College, Beijing, China; ^2^Key Laboratory for Conservation and Utilization of Bio-Resource, Key Laboratory for Microbial Resources of the Ministry of Education, School of Life Sciences, Yunnan Institute of Microbiology, Yunnan University, Kunming, China; ^3^College of Resources, Environmental Sciences and Chemistry, Chuxiong Normal University, Chuxiong, China; ^4^Chuxiong Medical and Pharmaceutical College, Chuxiong, Yunnan, China

**Keywords:** extremophiles, *Kocuria*, polyphasic taxonomy, comparative genomics, IAA production

## Abstract

Desert ecosystems have increasingly piqued the interest of microbiologists seeking novel bioactive compounds, as they are viewed as a largely uncharted reservoir of extremophiles with remarkable resilience to severe conditions. The genus *Kocuria*, belonging to the phylum *Actinomycetota*, is particularly notable for its documented capacity to flourish in such extreme environmental conditions. In this study, a total of 21 *Kocuria* strains were isolated from various ecosystems. Using polyphasic taxonomy approaches, eight strains from desert soils (CPCC 205273^T^, CPCC 205300, CPCC 205290, CPCC 205236^T^, CPCC 205293, CPCC 205292^T^, CPCC 205315^T^, CPCC 205268^T^) were identified representing five new species of the genus *Kocuria*. Strains CPCC 205281 and CPCC 205293 were identified as siblings of strain CPCC 205236^T^, while strains CPCC 205290 and CPCC 205300 were identified as siblings of strain CPCC 205273^T^. Additionally, *K. polaris* and *K*. *indcia* were determined to be the later heterotypic synonym of *K. rosea* and *K. marina*, respectively. Genomic analysis and physiological assays demonstrated that these previously uncharacterized strains were tolerant to high-level salt concentration and UV radiation, key survival traits in desert environments. The fermentation analysis revealed that most strains produced high-level contents of indole-3-acetic acid (IAA), although the complete gene sets for IAA biosynthesis were found in only one strain. Comparative genome analysis further showed that genes related to carbohydrate metabolism and transports were significantly enriched in desert-derived *Kocuria* strains, indicating adaptation to desert habitats. Collectively, our findings enhance our understanding of *Kocuria* taxonomy and highlight their genetic adaptation strategies to extreme environments, with potential biotechnological applications.

## 1 Introduction

Extremophilic and extremotolerant *Actinobacteria*, including acid-tolerant, alkalitolerant, psychrotolerant, thermotolerant, halotolerant, haloalkalitolerant, and xerophiles, are a group of bacteria that have been less well-studied (Sayed et al., 2020). Particularly, as the largest continental ecosystem on earth with approximately 30% of the total land area, deserts (Neilson et al., 2012; Publications Office of the European Union, 2018), have increasingly attracted the interest of microbiologists in the quest for novel bioactive compounds. *Actinobacteria* found in desert environments can thrive in specific circumstances of pH or salinity and possess impressive gene clusters that generally enable them to produce secondary metabolites with distinct antibacterial properties., making it a valuable subject of study (Bull et al., 2016; Idris et al., 2017; Cordero et al., 2018).

The genus *Kocuria*, a large group of actinobacteria, stand out for its known ability to survive in extreme conditions. The genus *Kocuria*, belonging to the family *Micrococcaceae*, the order *Micrococcales*, the phylum *Actinomycetota*, was first proposed by Stackebrandt et al. (1995) with *Kocuria rosea* as the type species. To date, *Kocuria* encompasses 26 species with validly published and correct names (data from LPSN).^1^ Members of the genus *Kocuria* are Gram-stain-positive, non-endospore-forming, catalase-positive, coagulase-negative, non-hemolytic cocci, and typically colonize distinct types of ecosystems such as marine, desert, rhizoplane, and hyper-saline soil (Kovacs et al., 1999; Kim et al., 2004; Mayilraj et al., 2006; Wang et al., 2015). Accordingly, they have been documented to produce biotechnologically interesting metabolites (Haidar et al., 2018; Retamal-Morales et al., 2018), auxins, pigments (Samanta et al., 2016; Sahar et al., 2022), extracellular polysaccharides (Kumar and Sujitha, 2014), and antibiotics (Palomo et al., 2013), under different, occasionally harsh, environments.

Indole-3-acetic acid (IAA), a phytohormone, plays a significant role in bacterial-plant interactions and has recently been identified as a key factor in bacterial stress response mechanisms (Duca and Glick, 2020). For example, IAA-producing strains like *Sinorhizobium meliloti* demonstrate increased resistance to environmental stressors, such as desiccation and UV radiation, by enhancing the production of protective compounds such as lipopolysaccharides (LPS), biofilms, extracellular polysaccharides (EPS), and trehalose (Bianco et al., 2009; Imperlini et al., 2009). Additionally, recent studies suggest a complex interaction between IAA synthesis and bacterial susceptibility to antibiotics, offering new insights into the physiological importance of IAA beyond its role in plant-microbe symbiosis (Cerboneschi et al., 2016; Chowdhury et al., 2016). Notably, some rhizosphric *Kocuria* strains are recognized as plant-growth-promoting rhizobacteria (PGPR) due to their IAA production (Karnwal, 2019; Pantoja-Guerra et al., 2023). While metagenomics analysis revealed that *Kocuria* was also the key and even dominant community of the plant-free desert soil (Vikram et al., 2016; Sun et al., 2018). This paradox suggests that IAA’s role in *Kocuria* might extend beyond plant-microbe interactions, potentially serving as a key mediator of bacterial stress tolerance in extreme environments.

In this study, we isolated and characterized 21 *Kocuria* strains collected from various semi-arid and hyper-arid environments, with a focus on eight strains from desert soils. Through polyphasic taxonomy and comparative genomics, we sought to classify these strains, investigate their genomic adaptations to extreme environments, and explore their capacity for producing IAA—a key trait for stress resistance and plant-microbe interactions.

## 2 Materials and methods

### 2.1 Samples collection and *Kocuria* strains acquisition

Samples used for bacterial isolation were mainly collected from deserts, rhizosphere soil in forests, and other diverse ecosystems. The soil samples were transported and stored at 4°C in the dark until further processing. According to the procedure described in previous study (Deng et al., 2022), the samples were subjected to gradient dilution and then spread on one-tenth strength R2A medium, supplemented with aztreonam (to the final concentrate of 25 mg l^−1^) and potassium dichromate (to the final concentrate of 50 mg l^−1^) to prevent or stymie the growth of Gram-stain-negative bacteria and fungi that may be present. The plates were subsequently incubated at 37°C for 3*–*4 weeks till the separated colonies appeared. Then the well-grown colonies were picked and transferred onto the freshly prepared R2A plates, and then purified by repeat streaking. The purified strains were maintained on aqueous glycerol suspensions (20%, v/v) at *−*80°C for a long time preservation.

Strain CPCC 205236^T^ and the other 11 strains were isolated from the desert soil samples collected from three different locations: Badain Jaran Desert (39°17′-39°31’N, 101°03′-103°00′E, 1,241–1,510 mH), Gurbantunggut Desert (44°48′-44°50’N, 88°16′-88°18′E, 600–620 mH), and Xinjiang Uygur Autonomous Region (43°13′-44°15’N, 84°45′-84°47′E, 2,620–2,637 mH). The remaining four soil-derived strains were obtained from the rhizosphere soil of an *Oxytropis falcata* located in Xinjiang province, China. In addition, four strains were recovered from the animal or human-associated ecosystems, including human skin and the feces of Crow. Only strain CPCC 204721 was isolated from the semi-arid air collected from Xinjiang province. Detailed information on the habitats of these newly isolated *Kocuria* strains was shown in Supplementary Table S1.

Strains *K. rosea* JCM 11614^T^ and *K. salina* DSM 28714^T^, which were used as references for partial parallel experiments, were obtained from the RIKEN BioResource Research Center (JCM) and German Collection of Microorganisms and Cell Cultures GmbH (DSMZ), respectively.

### 2.2 Phylogenetic analysis based on 16S rRNA genes

The genomic DNA of the strains in this study was extracted and their 16S rRNA genes were amplified by PCR, according to the method previously described by Li et al. (2007). The sequences were quality-checked and assembled using Seqman program (Swindell and Plasterer, 1997). The assembled sequences were then compared with available 16S rRNA gene sequences of cultured species from the EzBioCloud^2^ platform (Yoon et al., 2017). Multiple sequence alignments and phylogenetic analysis were conducted on the molecular evolutionary genetics analysis (MEGA) software package (version 7.0) (Kumar et al., 2016) using neighbor-joining (NJ) (Saitou and Nei, 1987) and maximum-likelihood (ML) (Felsenstein, 1981) methods. Confidences of the topology of trees were evaluated with the 1,000 bootstrap resamplings (Felsenstein, 1985).

### 2.3 Genome sequencing, assembly, and annotation

Whole-genome sequencing of the new isolates was conducted on the Illumina HiSeq 4,000 platform (Illumina, SanDiego, CA, USA) at the Beijing Genomics Institute (Beijing, China). Genomic DNA was randomly sheared to construct three read libraries of length 300 bp using a Bioruptor ultrasonicator (Diagenode, Denville, NJ, USA) and physicochemical methods. Paired-end fragment libraries were then sequenced using manufacturer protocols. Low-quality reads (those with consecutive bases covered by fewer than five reads) were discarded using Fastq v1.4 (Chen et al., 2018), and the remaining reads were assembled with the SOAPdenovo v1.05 software program (Xie et al., 2014). The quality (index: completeness and contamination) of the draft genomes was accurately assessed by the CheckM v1.2.3 pipeline (Parks et al., 2015). Assembled genomes with completeness > 80% and contamination < 5% were annotated by using Prokka v1.14.5 (Seemann, 2014) (All assemblies passed the quality control threshold: completeness > 80% and contamination < 5%). Genes related to stress response and IAA production were identified by comparison to the Uniprot^3^ and Interpro^4^ databases. Furthermore, biosynthetic gene clusters (BGCs) were detected and characterized using antiSMASH version 6.0.^5^ All parameters for the prior bioinformatic software were default ones unless stated otherwise.

### 2.4 OGRIs and phylogenomics analysis

The overall genome relatedness indices (OGRIs) were used to determine the similarity between genomes useful for species delineation (Chun and Rainey, 2014). Average nucleotide identity (ANI) and digital DNA–DNA hybridization (dDDH) values were calculated between the genomes of the newly isolated strains and their related species using the FastANI and the Genome-to-Genome Distance Calculator (GGDC, version 3.0)^6^ (Auch et al., 2010; Jain et al., 2018), respectively. To establish the phylogenomic relationships between the genus *Kocuria*, the single-copy orthologroups (SOG, one sequence per genome) were inferred by OrthoFinder v2.5.5 (Emms and Kelly, 2015, 2019) based on the annotated proteomes. For each SOG, a multiple sequence alignment was implemented using ClustalO v1.2.2 (Sievers et al., 2011), and the ambiguous sites were trimmed by trimAI v1.5.0 (Capella-Gutierrez et al., 2009) with the option “automated1.” A maximum-likelihood species tree was constructed based on a concatenation of these alignments using IQ-tree v2.3.6 (Nguyen et al., 2015; Minh et al., 2020) with 1,000 ultrafast bootstrap replicates, and the best model was selected by ModelFinder (Kalyaanamoorthy et al., 2017). In addition, MLSA (multilocus sequence analysis) was also implemented to evaluate the phylogenetic relationships. Forty conserved maker gene sequences were retrieved from genomes by using progenomes2 (Mende et al., 2019) with default options. Then, a phylogenetic tree was built using the same method as the phylogenomics based on the SOGs.

### 2.5 Growth conditions, morphological characteristics, and physiological tests

Physiological characteristics of strains CPCC 205293, CPCC 205236^T^, CPCC 205292^T^, CPCC 205300, CPCC 205315^T^, CPCC 205268^T^, CPCC 205273^T^, CPCC 205290, and two reference strains were determined following growth on various media, including on peptone yeast (PYG; 3% trypticase soy broth, 0.3% yeast extract, 1.5% agar; pH 7.0–7.2), tryptone soy agar (TSA), International Streptomyces Projects 2 medium (ISP 2), yeast extract sucrose (YM), Luria-Bertani, (R2A), International Streptomyces Projects 4 medium (ISP 4), and nutrient (Shirling and Gottlieb, 1966) media formulations, after incubation at 28°C for 1–2 weeks. The growth conditions at different temperatures were assayed in TSA medium at temperatures of 4, 10, 20, 26, 28, 30, 32, 37, 40, 45, 46, and 50°C for 14 d (Sun et al., 2017). The range of pH for growth (4.0–11.0 with 1 interval) was tested on TSA with a previously described buffer system (Xu et al., 2005). Salt (sodium chloride) tolerance was determined on TSA agar medium plates supplemented with NaCl concentrations of 0, 1, 2, 3, 4, 5, 6, 7, 8, 9, 10, 15, and 20% (w/v). UV (Ultraviolet) radiation tolerance was tested according to the previously described procedures (Zhang et al., 2007). Growth after 10 days was identified as positive or negative relative to un-irradiated controls.

Gram staining reaction of the isolates was conducted as the method previously described (Magee et al., 1975). Colony appearance and pigment production were observed after incubation at 28°C on TSA medium. Cellular morphologies were observed by light microscopy (Zeiss Axio Scope. A1 Vario) and transmission electron microscopy (JEOL JEM-1010) after incubation for 5–7 days. The motility of cells was observed by light microscopy by first growing the strain on TSA semi-solid medium agar (0.3%, *w/v*). Oxidase activity was identified using an analytical profile index (API) oxidase reagent (bioMeriéux) according to the manufacturer’s instructions. Catalase activity was determined by bubble production in 3% (v/v) H_2_O_2_. Metabolic characteristics were determined using Biolog GEN III (MicroPlate), API 50CH, API 20NE, and API ZYM (bioMerieux) test kits following the manufacturer’s instructions. The tests for the formation of indole and H_2_S and the hydrolysis of gelatin, cellulose, and starch, were performed as previously described by Gonzalez et al. (1978).

### 2.6 Chemotaxonomic assays

Chemotaxonomic and molecular systematic studies of these eight isolates and reference strains were conducted with cells after cultivation in TSB (tryptone soy broth) medium at 28°C in shake flasks on a rotary shaker (150 r/min) until cells reached the logarithmic growth phase. Amino acids and peptides in whole-cell hydrolysates were analyzed by two-dimensional ascending thin-layer chromatography (TLC) on cellulose plates using solvent systems described by Schleifer and Kandler (1972). Sugar profiles were evaluated with TLC, as previously described by Komagata and Suzuki (1988). Analysis of respiratory quinones was carried out by the Identification Service of DSMZ (Braunschweig, Germany). Menaquinones were extracted according to Collins et al. (1977) and analyzed by High Performance Liquid Chromatography (HPLC) (Groth et al., 1997). Cellular fatty acids analysis was performed using the Microbial Identification System (Sherlock Version 6.0; MIDI database: ACTIN1) (Kroppenstedt, 1985; Sasser, 1990). Polar lipids were extracted and identified by two-dimensional TLC following the methods of (Minnikin et al., 1984).

### 2.7 Quantification of IAA synthesis

The bacterium culture was maintained at 30°C in LB medium supplemented with 120 mM methanol for 8 days at 200 rpm. Spectrophotometric quantification of indole-3-acetic acid (IAA) was evaluated by using Salkowski reagent (Ehmann, 1977). In brief, culture filtrate was collected by centrifugation at 7,000 rpm for 5 min. 0.5 mL aliquots of culture was reacted with 2 mL of Salkowski reagent [2% (w/v) FeCl₃·6H₂O in 35% (v/v) HClO₄], followed by the addition of 200 μL orthophosphoric acid (H_3_PO_4_, 85%) under vortex mixing. The reaction mixture was incubated in darkness for 30 min at ambient temperature (25 ± 1°C). Absorbance measurements were conducted at 530 nm using a UV–Vis spectrophotometer (Agilent Technologies, USA), with quantification achieved through a standard calibration curve generated from pure IAA (0–200 μg/mL; Fisher Scientific, Hampton, NH, USA). The experiment was repeated three times. The mean values of repetitions were calculated ± standard deviation (SD).

### 2.8 Comparison of functional genes

In this study, strains from desert-associated ecosystems were designated as DAK strains, whereas those from other ecosystems were designated as NDAK strains. To perform comparative analysis for desert-associated *Kocuria* strains (DAK) and other non-desert-associated *Kocuria* strains (NDAK), we constructed a genomic dataset consisting of 179 individuals affiliated to the genus *Kocuria* (21 strains were from this analysis and others were collected from the NCBI assembly database). Among which 25 strains with uncertain isolation sources were excluded. Genomes from the same strain with an ANI greater than 99.95% and alignment fraction exceeding 50% were marked as redundant genomes, and then they were dereplicated by filtering out one of the genomes at random. Finally, the remaining 143 genomes formed a representative *Kocuria* genome dataset (Supplementary Table S2). The comparison of functional genes between DAK and NDAK was performed according to the previous study (Levy et al., 2017). A random set with 15 genomes of NDAK was selected, and the mean and s.d of the phylogenetic distance in the set were calculated. This step was repeated 150 times, and then these 15 genomes of NDAK with minimum differences between their mean and s.d. of phylogenetic distances were selected for subsequent analysis. Open reading frames (ORFs) of these genomes were predicted using Prokka v1.14.5. The amino acid sequences of these putative ORFs were aligned against Cluster of Orthologous Groups (COG) and Kyoto Encyclopedia of Genes and Genomes (KEGG) databases to obtain their corresponding annotations using eggnog Mapper v5.0 with default options (Huerta-Cepas et al., 2019). Phylogenetically informed principal component analysis (phylo-PCA) was used to visualize the differences in gene compositions between DAK and NDAK strains using the R package phytools v0.7–80 (Revell, 2024). The pan-genome-wide association studies (pan-GWAS) analysis was performed for identification of habitat-enriched KEGG orthogroups (KOs) using SCOARY v1.6.16 (Brynildsrud et al., 2016) depending on the KO presence/absence data set. Significant variants were determined using a cutoff of Bonferroni adjusted *p* < 0.05 with sensitivity and specificity greater than 80%.

## 3 Results and discussions

### 3.1 Identification of *Kocuria* spp.

Twenty-one strains were isolated from the desert soils, human skin, feces, and air samples from China (Supplementary Table S1). Comparative analysis of their 16S rRNA gene sequences revealed that these newly isolated strains belong to the family *Micrococcaceae* and were closely related to the genus *Kocuria*.

The comparative analysis of 16S rRNA gene sequences indicated that these newly isolated strains were members of the family *Micrococcaceae* with closely related to the genus *Kocuria*. Thirteen strains, including CPCC 205231, CPCC 205263, CPCC 205258, CPCC 205274, CPCC 205281, CPCC 205293, CPCC 205236^T^, CPCC 205273^T^, CPCC 205290, CPCC 205300, CPCC 205268^T^, CPCC 205315^T^, and CPCC 205292^T^, exhibited high 16S rRNA gene sequence similarities to *K. polaris* CMS 76or^T^ (99.1–99.6%), *K. rosea* DSM 20447^T^ (99.2–99.8%), *K. himachalensis* K07-05^T^ (99.3–99.9%), and *K. salina* DSM 28714^T^ (99.1–99.9%). Stains CPCC 204721, CPCC 205233, CPCC 205261, and CPCC 205297 shared the highest 16S rRNA gene similarity with *K. indica* NIO-1021^T^ (99.86–99.93%). Strain CPCC 205235 showed the 99.7% similarity with *K. carniphila* CCM 132^T^; CPCC 104605 showed 100% identity with *K. subflava* YIM 13062^T^; while CPCC 205260 and CPCC 205295 shared 100% 16S rRNA gene sequence identity with the *K. palustris* DSM 11925^T^.

**Figure 1:**
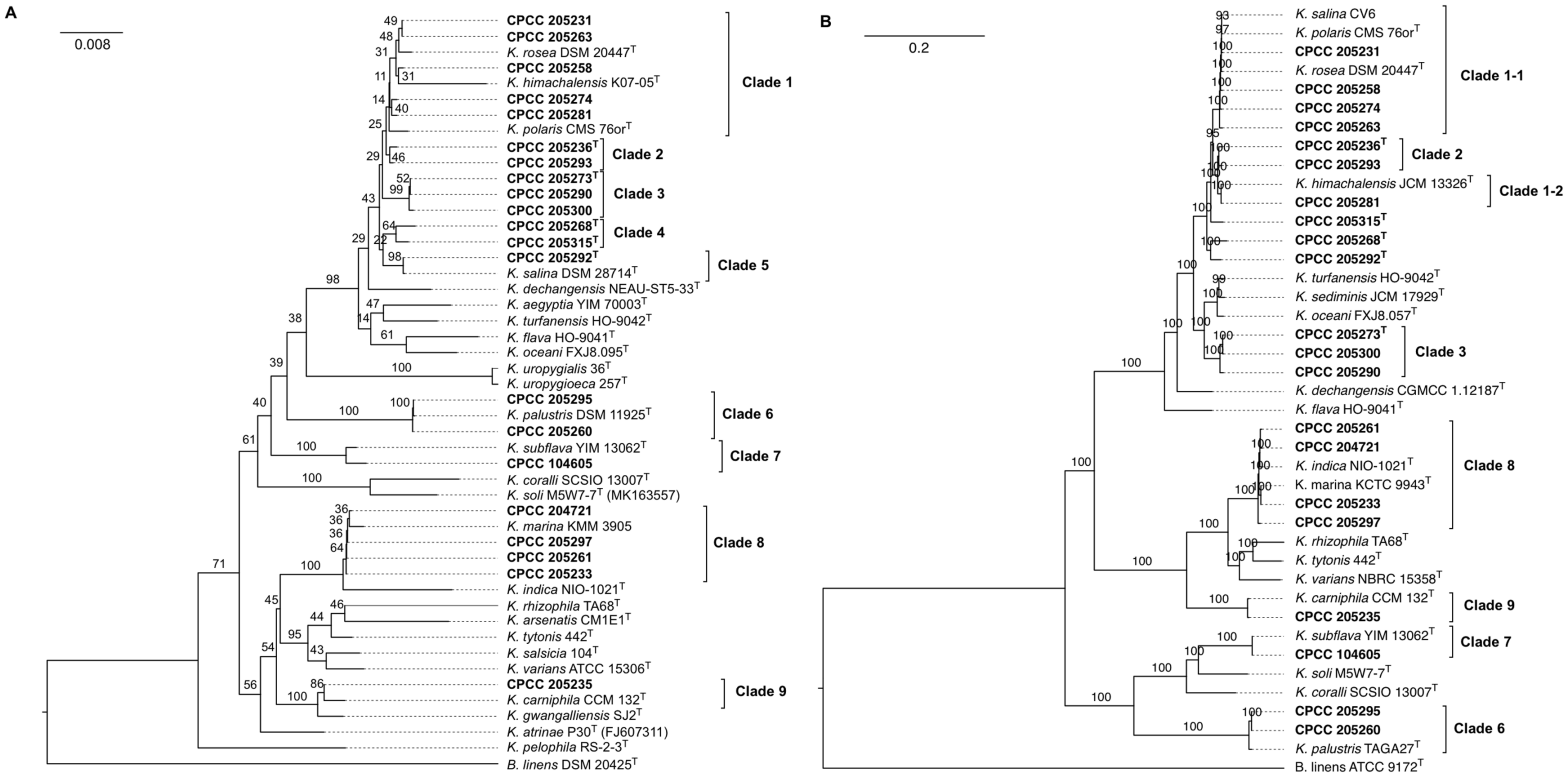
The phylogenetic analysis based on 16S rRNA gene **(A)** and core genome **(B)** of 21 newly isolated strains and 26 type strains of established species among the genus *Kocuria*. Strains isolated from this study are shown in bold. Bootstrap values that more than 70% are indicated for each node (1,000 replicates). *Brevibacterium linens* DSM 2042^T^ was used as an outgroup.

The 16S rRNA gene sequences of these 21 strains isolated from this study and 26 validated described type strains with correct names (Data from LPSN) were used to construct the phylogenetic trees. The phylogenetic tree based on the NJ method showed that these newly isolated strains fall into nine distinct phylogenetic groups denoted as Clade 1–9 (Figure 1A). However, Clades 1, 2, and 4 showed ambiguous phylogenetic placements between NJ and ML trees with low bootstrap values (Supplementary Figure S1). This ambiguity may be attributed to the high homogeneity of the 16S rRNA gene sequences within these clades, making it challenging to differentiate between strains using a single gene.

### 3.2 Genome-based taxonomy

To further confirm the phylogenetic positions of these *Kocuria* strains, their genome sequences were determined and then subjected to phylogenomic tree reconstruction and MLSA with other reference genomes (Supplementary Table S1) (*Brevibacterium linens* DSM 2042^T^ was used as the outgroup). Firstly, 40 universal markers were retrieved from these genomes, and aligned and concatenated to build an MLSA-based phylogeny. In addition, 662 single-copy orthogroups were inferred by OrthoFinder and then used to reconstruct a phylogenomic tree. Interestingly, phylogenomics and MLSA-based phylogeny provide a consistent and reliable phylogenetic topology (Supplementary Table S2; Figure 1B), especially on the internal ancestral nodes of the lower support phylogenetic groups recovered from the 16S rRNA-based phylogeny. Clade I with the lowest bootstrap values in 16S rRNA-based phylogeny segregated into two subclade (Clade 1–1 and Clade 1–2) with higher support inside the MLSA and phylogenomic trees. Strain CPCC 205281 and *K. himachalensis* JCM 13326^T^ of Clade 1 formed a subclade (Clade 1–1), and it was distant from the other members of that clade (Figure 1B). The strain CPCC 205268^T^ of Clade 4 were clustered with CPCC 205292^T^ of Clade 5 with a bootstrap value of 100%, whereas another member of Clade 5 (*K. salina* CV6) was distributed in the Clade 1–1. In addition, CPCC 205215^T^ was placed independently of the other *Kocuria* species and formed a monophyletic clade with a bootstrap value of 100%. The phylogenetic placements of the other six groups (Clade 2, 3, 6–9) were utterly consistent with that in the 16S rRNA gene tree.

The overall genome relatedness indices (OGRIs) values of 21 strains isolated from this study with their closely related strains were calculated by algorithms fastANI and GGDC. The strains within two subclades (Clade 1–1 and Clade 1–2) and six clades (Clade 2, 3, 6–9) exhibited dDDH and ANI values exceeding the suggested threshold for species delineation values of 70% for dDDH and 95–96% for ANI (Chun et al., 2018; Riesco and Trujillo, 2024), respectively, while the values between each subclade and/or clade were below these cut-off values (Supplementary Figures S3, S4). The ANI and dDDH values between strain CPCC 205268^T^ and other *Kocuria* strains involved in this study were in a range of 78.5–91.3% and 20.1–41.7% (Supplementary Figures S3, S4), which were lower than the minimal threshold for species delineation. The same situation occurred between CPCC 205215^T^ (ANI = 78.1–93.4%, dDDH = 20.0–51.3%) and CPCC 205292^T^ (ANI = 87.1–92.4%, dDDH = 20.0–43.7%). The ANI and dDDH values for the studied strains were consistently below the 95–96 and 70% thresholds, respectively, confirming their distinction as new species.

Consequently, OGRIs combined with phylogenomics, the phylogeny of 16S rRNA gene indicated that 8 of 21 *Kocuria* strains (CPCC 205273^T^, CPCC 205290, CPCC 205300, CPCC 205268^T^, CPCC 205315^T^, CPCC 205292^T^, CPCC 205293, and CPCC 205236^T^) isolated from this study represent five new species, and others belong to the previous established species. In addition, genome-based taxonomic characterization demonstrated that *K. polaris* should be considered as a later heterotypic synonym of *K. rosea* and *K. indica* that as of *K. marina*, respectively.

### 3.3 Morphological, physiological, and biochemical features

The phenotypic properties of these eight newly isolated strains were compared with their closely related type strains to acquire a deeper understanding of their relationships. All strains were Gram-strain-positive, oxidase-negative, catalase-positive, non-motility, and aerobiotic coccoid. The key phenotypic characteristics of these eight isolates were compared with those of their closely related species in Table 1. Strain CPCC 205273^T^, CPCC 205290, and CPCC 205300 could be differentiated from *K. rosea* DSM 20447^T^ by their positive alkaline phosphatase activity, while they did not grow at more than 50°C, and did not utilize D-galactose and D-melibiose. Strain CPCC 205236^T^ and CPCC 205293 were positive for alkaline phosphatase but negative for cystine arylamidase; they could not grow at 50°C and utilize D-melibiose, and D-galactose, which distinguished them from *K. rosea* DSM 20447^T^. Differentials between CPCC 205315^T^, CPCC 205268^T^, and *K. rosea* DSM 20447^T^ were hydrolysis of starch and assimilation of L-alanine. Moreover, CPCC 205292^T^ was distinguished from *K. salina* DSM 28714^T^ by its hydrolysis of cellulose and assimilation of L-alanine.

**Table 1:** Differentiating characteristics between the newly isolated strains and the related *Kocuria* reference strains.

Characteristics	1	2	3	4	5	6	7	8	9	10
Isolation source	Desert Sand	Desert Rhizosphere	Desert Sand	Desert Sand	Desert Sand	Desert Sand	Desert Sand	Unknown	Desert Sand	halophyte Rhizosphere
Colony color	Pale orange	Pale orange	Orange	Pale orange	Pale orange	Pale orange	Orange	Orange	Pastel orange	Pastel orange
NaCl tolerance (%)	0–10	0–15	0–20	0–15	0–10	0–10	0–10	0–10	0–10	0–10
Temperature (Optimal) (°C)	4–37 (26–32)	4–42 (26–32)	4–40 (26–32)	4–35 (26–32)	4–40 (26–32)	4–35 (26–32)	4–35 (26–32)	4–52 (26–32)	4–42 (26–32)	4–40 (26–32)
pH range (Optimal)	6.0–11.0 (7.0–9.0)	6.0–11.0 (7.0–9.0)	7.0–11.0 (7.0–9.0)	6.0–11.0 (7.0–9.0)	6.0–11.0 (7.0–9.0)	6.0–11.0 (7.0–9)	6.0–11.0 (7.0–9.0)	6.0–11.0 (7.0–9.0)	7.0–11.0 (7.0–9.0)	6.0–11.0 (7.0–9.0)
Hydrolysis of										
Cellulose	−	+	+	−	−	−	−	−	+	−
Starch	+	+	+	+	+	+	+	−	+	+
Enzyme activities										
Acid phosphatase	−	−	−	−	−	−	+	−	−	−
Alkaline phosphatase	+	+	+	+	+	+	+	−	−	+
Cystine arylamidase	+	−	−	−	−	−	−	+	+	−
Esterase lipase (C8)	+	+	+	+	+	+	+	+	+	+
Utilization of										
D-Arabitol	+	−	−	+	−	+	+	−	+	−
D-Cellobiose	+	+	+	+	+	+	+	+	+	−
D-Galactose	−	−	−	−	−	+	−	+	+	+
D-Melibiose	−	−	−	−	−	−	−	+	−	−
D-Raffinose	−	−	−	−	−	−	−	−	+	+
D-Sorbitol	+	+	+	+	−	+	+	+	+	−
L-Alanine	+	+	−	+	−	+	+	−	+	+
L-Arginine	−	+	−	+	−	+	−	+	−	+
*α*-D-Lactose	−	−	−	−	−	−	−	+	−	−
D-Salicin	+	+	+	−	+	+	+	+	−	−
DNA G + C (%)	69.2	72.3	69.0	72.2	68.2	71.8	71.8	72.8	72.8	72.2
Major fatty acids (>10%)	anteiso- C_15:0_ (64.1%), iso-C_15:0_ (15.1%)	anteiso- C_15:0_ (49.2%), iso-C_15:0_ (31.6%)	anteiso- C_15:0_ (59.3%), C_16:1_*ω*7*c* (11.3%)	anteiso- C_15:0_ (56.5%), iso-C_15:0_ (13.0%), C_16:1_*ω*7*c* (10.6%)	anteiso- C_15:0_ (57.5%), iso-C_15:0_ (15.4%), C_16:1_*ω*7*c* (10.8%)	anteiso- C_15:0_ (55.4%), C_16:1_*ω*7*c* (14.6%), iso-C_15:0_ (11.2%)	anteiso- C_15:0_ (53.1%), iso-C_15:0_ (13.0%), C_16:1_*ω*7*c* (11.5%)	anteiso- C_15:0_ (59.9%), iso-C_15:0_ (10.4%)	anteiso- C_15:0_ (50.5%), C_16:0_ (14.9%)	anteiso- C_15:0_ (41.3%), iso-C_15:0_ (18.6%)
Menaquinone(s)	MK-8(H_2_):MK-7(H_2_): MK-9(H_2_) = 80:12:8	MK-8(H_2_):MK-7(H_2_): MK-9(H_2_) = 78:15:7	MK-8(H_2_):MK-7(H_2_): MK-9(H_2_) = 78:14:8	MK-8(H_2_):MK-7(H_2_): MK-9(H_2_) = 83:11:6	MK-8(H_2_):MK-9(H_2_):MK-7(H_2_) = 74:18:8	MK-8(H_2_):MK-9(H_2_):MK-7(H_2_) = 73:19:8	MK-8(H_2_):MK-9(H_2_):MK-7(H_2_) = 72:19:9	MK-8(H_2_):MK-7(H_2_):MK-9(H_2_) = 61:33.4:5.6	MK-8(H_2_): MK-7(H_2_): MK-9(H_2_) = 76:14:10	MK-8(H_2_):MK-7(H_2_):MK-9(H_2_) = 71:11.7:10.6

Strains: 1, CPCC 205315^T^; 2, CPCC 205268^T^; 3, CPCC 205273^T^; 4, CPCC 205290; 5, CPCC 205300; 6, CPCC 205236^T^; 7, CPCC 205293; 8, *K. rosea* JCM 11614^T^; 9, CPCC 205292^T^; 10, *K. salina* DSM 28714^T^. All data were from this study. Symbols: +, positive; −, negative.

The major polar lipids of these eight strains were diphosphatidylglycerol (DPG), and phosphatidylglycerol (PG), which is consistent with those of other *Koruria* species (Supplementary Figure S5). The *anteiso*-C_15:0_ was the predominant cellular fatty acid in all of these strains, in line with that of other members of the genus *Kocuria*. The other fatty acid compositions were detected in each strain but varied (Table 1; Supplementary Table S3). All strains contained MK-7(H_2_), MK-8(H_2_), and MK-9(H_2_) in their respiratory quinone systems. Quantitative differences in quinones between these previously uncharacterized strains and type strains of closely related species are given in Table 1.

On the basis of phylogenetic analysis, genomic relatedness, and phenotypic feature comparison, it was proposed that eight newly isolated strains should be classified as five new *Kocuria* species. The formal proposal of these new species is in the *Species Descriptions* section.

IAA (indole-3-acetic acid), a product of the amino acid L-tryptophan (L-Trp) (Yu et al., 2017), acts as a plant growth regulator. Several studies have revealed that a wide variety of bacteria have been able to produce the IAA such as *Agrobacterium tumefaciens*, *Pseudomonas syringae*, *Streptomyces* sp., *Bacillus subtilis* spp., *Pseudomonas fluorescens*, and *Bacillus megaterium* (Rushabh et al., 2020). IAA was detected and quantified in the fermentation broth of the eight newly isolated strains and two reference strains. Eight out of ten strains showed the ability to convert L-Trp into IAA. Quantification analysis revealed that the IAA content yield from the fermentation broth of CPCC 205236^T^, CPCC 205292^T^, CPCC 205293, CPCC 205290, CPCC 205315^T^, CPCC 205268^T^, *K. rosea* JCM 11614^T^, and *K. salina* DSM 28714^T^ was 126.9 ± 5.7, 256.8 ± 8.9, 151.3 ± 4.3, 148.0 ± 7.9, 177.8 ± 0.35, 200.8 ± 3.4, 86.4 ± 5.2, and 257.6 ± 6.2 mg/L, respectively (Supplementary Figure S6).

### 3.4 Genome-wide screen for key genes associated with adaptation to extreme environments among *Kocuria* strains

**Figure 2:**
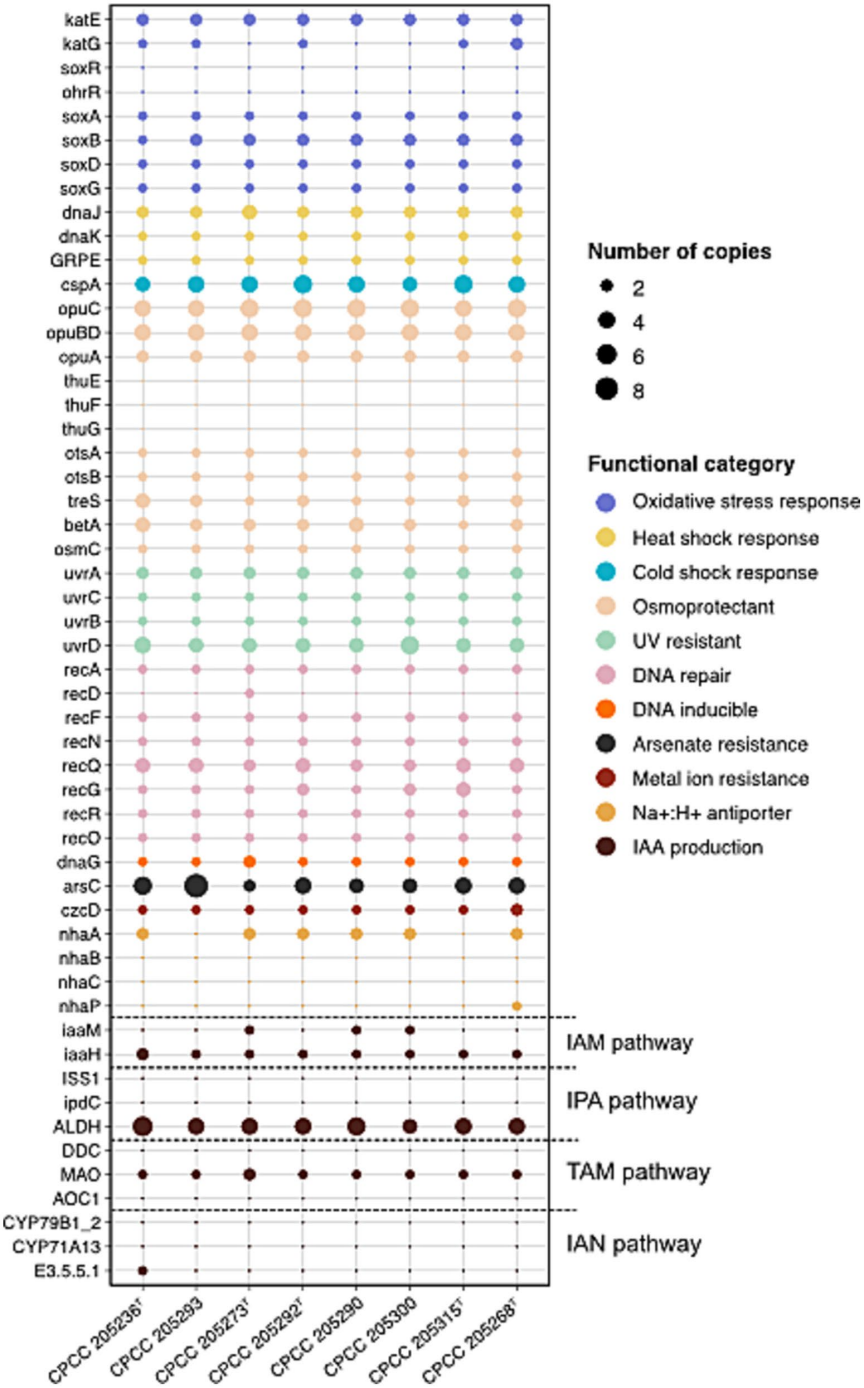
The distribution of genes involved in stress response and IAA production pathways in eight *Kocuria* strains isolated from desert soils.

Bacteria in the desert are subjected to a variety of physicochemical stresses, including low organic carbon and nitrogen availability, high UV irradiation, dryland salinity, and temperature extremes, microbes cope with these environmental stresses through unique physiological acclimation mechanisms (Georgiou et al., 2015; Allison, 2023). Therefore, the genes linked to stress response (Supplementary Table S4) were discovered in the genomes of the eight previously uncharacterized *Kocuria* strains isolated from the desert soil (Figure 2). Eight desert-derived *Kocuria* strains harbored high-abundance genes for the Opu transporter (*opuABCD*) and trehalose biosynthesis (*otsAB* and *treS*), both linked to osmoprotection under desiccation stress (Rath et al., 2020; Lee et al., 2023). Genes encoding heat (*dnaJ*, *dnaK*, and *GRPE*) and cold (*cspA*) shock proteins (Szabo et al., 1994) were also conserved across strains, supporting their adaption to extreme temperature fluctuations. Bacteria’s primary antioxidant defense systems, catalase (*katE*) enzymes (Lushchak, 2001), may not protect them from extreme and prolonged oxidative stress, leading them to activate SoxRS systems in response to redox-active compounds (Nunoshiba et al., 1993). Interestingly, all strains exhibited *katE* and the full set of genes associated with the SoxRS system (*soxABDR*). All strains contained *uvrABCD* and *recADFNQG*, which were separately involved in NER (nucleotide excision repair) mediated UV-radiation resistance and RecA-mediated repair of DNA double-strand breaks. Our observations suggested that these studied *Kocuria* strains isolated from the desert soil have a complete genetic basis for survival in extreme environments, like hyper-arid deserts.

In stress response mechanisms, IAA emerges as a key player in bacterial survival under extreme environmental conditions. Studies on *Sinorhizobium meliloti* have shown that the IAA-overproducing mutant exhibits enhanced resilience to UV irradiation, acidity, osmotic shock, and heat shock in comparison to the wild-type strain (Imperlini et al., 2009). In microorganisms, the L-Trp-dependent IAA biosynthesis is divided into five pathways according to the different intermediate metabolites: indole-3-acetamide (IAM), indole-3-pyruvic acid (IPA/IPyA), indole-3-acetonitrile (IAN), tryptamine (TAM), and TSO (L-Trp side-chain oxidase) pathways. Since the mechanism of the TSO pathway remains unknown, we excluded it and explored a pool of genes involved in the other four L-Trp-dependent pathways (Supplementary Table S4; Figure 2). Out of these four routes, the IAM pathway showed the most conserved genetic architecture in genomes of eight newly described *Kocuria* strains. While *iaaH* (encoding indoleacetamide hydrolase) was universally present, the key *iaaM* (encoding tryptophan-2-monooxygenase) was exclusively detected in genomes of strain CPCC 205273^T^, CPCC 205290, and CPCC 205300. Intriguingly, domain analysis (via InterProScan with default options) revealed uncharacterized sequences harboring a catalytic core domain of *IaaM* (Amino_oxidase) across all strains, suggesting the existence of *IaaM* isozymes with analogous functions. In addition, other actinobacteria phylogenetically related to *Kocuria* predominantly utilized the IAM pathway for IAA synthesis, supported by conserved iaaM/iaaH clusters (Lin and Xu, 2013; Myo et al., 2019). Therefore, these observations implied that these *Kocuria* strains employed the IAM pathway for IAA production, potentially relying on isozymes to compensate for incomplete *iaaM* in certain strains.

### 3.5 Secondary metabolite biosynthesis gene clusters

Actinobacteria, particularly those found in extreme habitats, possess a diverse range of unique bioactive chemicals through their secondary metabolism, which may have significant medical applications. Here, using antiSMASH, the secondary metabolite BGCs of these desert-associated previously uncharacterized strains were identified based on their genome sequence. Eight *Kocuria* strains exhibited six to nine BGCs with moderate similarities to previously described secondary metabolite biosynthetic gene clusters (Supplementary Table S5). These BGCs exhibited 5–100% similarities to documented secondary metabolite biosynthetic gene clusters including those for carotenoid, dactylocycline A, desferrioxamin B, microansamycin, stenothricin, glycopeptidolipid, *ε*-poly-L-lysine, oxalomycin B, salinipeptin A/B/C/D, FW0622 and branched-chain fatty acids. In addition, other unidentified secondary metabolite clusters were predicted that were attributable to those encoding NAPAA (Non-alpha poly-amino acids like e-Polylysin), RiPP-like (Post-translationally modified peptide like) compounds, indole, NRPS-like (Non-ribosomal peptide synthetase like) compounds, T3PKS (Type III polyketide synthases), linaridin, NI-siderophore, and terpene types (Supplementary Table S5).

### 3.6 Functional genes enrichment in desert-derived *Kocuria* strains

**Figure 3:**
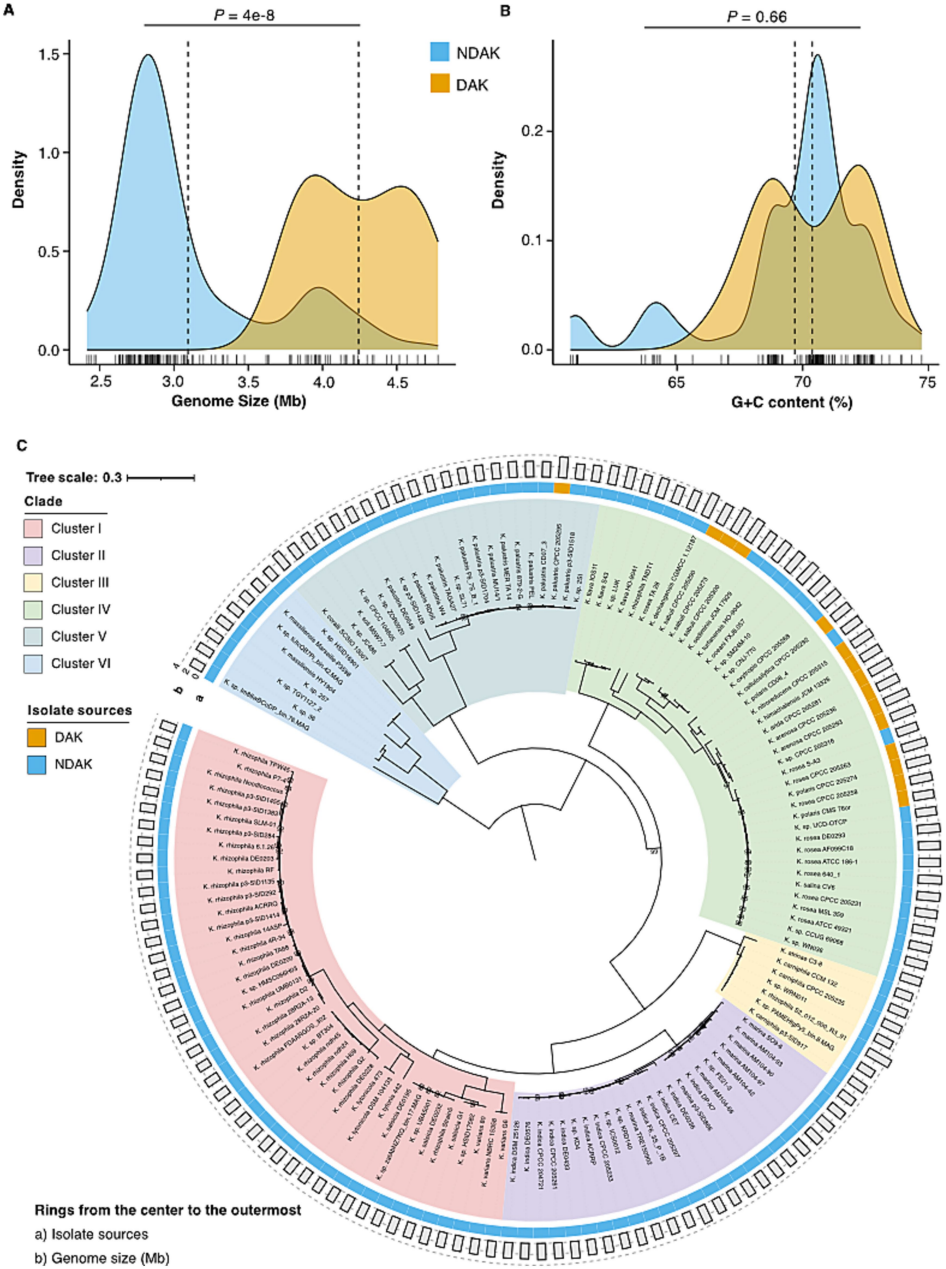
Genomic features comparison and phylogenomics of *Kocuria* strains. **(A)** The comparisons of genomic size and G + C content between DAK and NDAK strains. **(B)** The comparisons of G+C content between DAK and NDAK strains. **(C)** The phylogenetic tree based on the genome sequences of 143 *Kocuria* strains. Rings outside the tree are depicted by isolated sources (ring a) and genomic size (ring b). Bootstrap values that are equal to 100% are not shown. The scale bar denotes 0.3 substitutions per amino acid position.

To decipher the underlying mechanism of desert adaptation among *Kocuria*, we conducted a comparative analysis for desert-associated *Kocuria* strains (DAK) and other non-desert-associated *Kocuria* strains (NDAK). The genome size of DAK was 4.24 Mb ± 0.51 on average, which was significantly larger than that of NDAK (3.09 Mb ± 0.34) (Mann–Whitney *U* test, *p* = 4e-8) (Figure 3A). However, the G + C content has no difference between DAK (70.4% ± 2.02%) and NDAK (69.7% ± 2.99%) (Mann–Whitney *U* test, *p* = 0.66) (Figure 3B). Based on 207 SOGs, we performed a phylogenomic analysis among the 143 *Kocuria* strains from different habitats to estimate their evolutionary relationships. A maximum-likelihood species tree showed that these *Kocuria* strains diverged into six clusters labeled as Cluster I-VI, respectively (Figure 3C). All of the DAK strains fell into Cluster IV except for CPCC 205295. Interestingly, by evaluating the size of their genomes, we found that the strains with relatively larger genomes were also mainly clustered into Cluster IV. It implied that the *Kocuria* strain might exhibit genomic expansion as a concomitant response to its adaptation to the desert environment.

**Figure 4:**
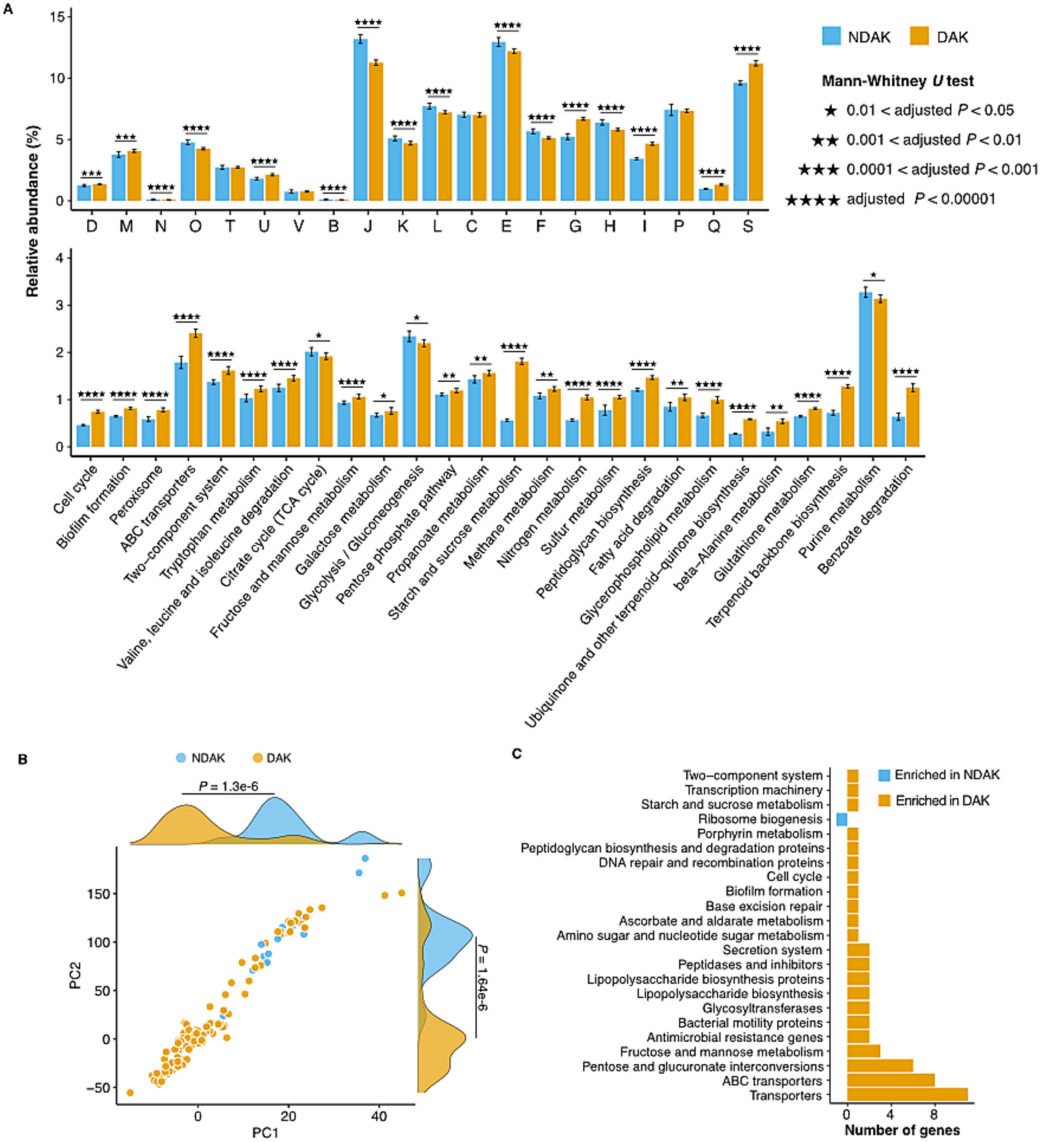
Enrichment analysis of functional categories in DAK and NDAK strains. **(A)** Pairwise comparison of core genomes among DAK and NDAK strains based on COG (top panel) and KEGG database (bottom panel). Bar charts represent the relative abundance of gene categories in statistical tests, and the error bars represent the standard deviations of relative abundance of categories in the core genomes of DAK or NDAK strains. **(B)** Phylogenetically informed principal-component analysis (phylo-PCA) of the KEGG pathways between DAK and NDAK strains. **(C)** Pan-GWAS analysis results showing typical KOs associated with desert environments by using SCOARY.

The comparison of the core genome functions between DAK and NDAK strains was performed according to the methods of a previous study (See Methods). The results based on the COG database show that COG categories involved in the Intracellular trafficking, secretion, and vesicular transport (COG U), Cell wall/membrane/envelope biogenesis (COG M), carbohydrate transport and metabolism (COG G), and lipid transport and metabolism (COG I) were enriched in the DAK strains (Figure 4A top panel; Supplementary Table S6). As for the KEGG database, 21 pathways were significantly different between DAK and NDAK strains, and many of these metabolic pathways were related to environmental adaptation, including ABC transports, two-component system, and energy metabolism pathways (Figure 4A bottom panel; Supplementary Table S7) (Aujoulat et al., 2012; Ma et al., 2021). In particular, the relative abundance of starch and sucrose metabolism of DAK strains was two times the level of NDAK.

Genome-wide differences investigation of the KEGG pathways between DAK (15 strains) and NDAK (128 strains) strains was performed by phylogenetically informed principal component analysis (phylo-PCA) and pan-genome-wide association studies (Pan-GWAS) based on the presence/absence data of KOs. Firstly, phylo-PCA analysis shows that KEGG pathways in DAK and NDAK strains were divided into two clusters, which were significantly different (Mann–Whitney *U* test, *p* < 0.05) on both the PC1 and PC2 axes (Figure 4B), suggesting the distinct functional composition among DAK and NDAK strains. In addition, Pan-GWAS identified 30 KOs that were positively associated with desert habitat with an adjusted *p* value less than 0.05 (Figure 4C; Supplementary Table S8). The majority of these enriched KOs were assigned to the pathways of environmental signal response (transports and ABC transports) and carbohydrate metabolism (fructose and mannose metabolism and pentose and glucuronate interconversions), suggesting the major role in the desert adaptation of *Kocuria* strains.

## 4 Conclusion

In this study, we isolated and identified 21 *Kocuria* strains from diverse ecosystems. Based on the polyphasic taxonomic approaches, strains CPCC 205236^T^ (together with CPCC 205293), CPCC 205273^T^, CPCC 205292^T^ (together with CPCC 205290 and together with CPCC 205300), CPCC 205315^T^, and CPCC 205268^T^ were verified to represent five new species of the genus *Kocuria*. Phenotypic and genomic analysis of these new strains demonstrated that the adaptation to desert niches may be achieved through various response strategies to UV radiation, carbon starvation, desiccation, and osmotic stress. Most of these newly isolated strains (6/8, 75%) could convert L-Trp to IAA by qualitative analysis of the fermentation, despite genome analysis revealing that only one of them processed the potential of IAA production. Moreover, the comparative genomics analysis revealed that the genes related to carbohydrate metabolism and transporters have been enriched in desert-associated *Kocuria* strains. Our findings underscored the critical role of *Kocuria* organisms in hyper-arid deserts and uncovered several genomic traits for adaption to the desert environment.

## 5 Taxonomic descriptions

### 5.1 Species descriptions

#### 5.1.1 *Kocuria arenosa* sp. nov.

*Kocuria arenosa* (a.re.no’sa. L. fem. Adj. arenosa, sandy, dwelling in desert sand).

Cells are Gram-stain-positive, aerobic, coccoid (0.8–1.2 μm in diameter), non-motile, non-spore-forming. Colonies are 0.8–2.0 mm in diameter, convex with smooth, round, uniformly edged, opaque and pastel orange (sometimes pale orange or orange) in color on TSA medium. Growth temperature and pH range for growth are 4–42°C and pH 6.0–11.0, with optima growth at 26–32°C and pH 7.0–9.0. Can tolerate up to 10% (w/v) NaCl. Catalase-positive and oxidase-negative. Hydrolysis of starch are positive, but negetive for hydrolysis of gelatin and cellulose. Assimilation of D-arabitol, D-cellobios, D-, D-sorbitol, L-alanine, Inosine, gentiobiose, and salicin. Cells are positive for alkaline phosphatase, esterase lipase (C8), and *β*-glucosidase, but negetive for cystine arylamidase and β-galactosidase. IAA is detected from the fermentation broth. The polar lipids profile mainly contains diphosphatidylglycerol and phosphatidylglycerol. The major fatty acids (>10%) consist of anteiso-C15:0, C_16:1_ *ω*7*c* and iso-C_15:0_. The predominant quinone is MK-8(H_2_), with minor of MK-7(H_2_) and MK-9(H_2_).

The type strain, CPCC 205236^T^ (= KCTC 59060^T^), was isolated from sand soil in Gurbantunggut Desert, China. The genome size is 4.2 Mbp, and the genomic DNA G + C content is 71.8%. The 16S rRNA gene and whole genome sequence of strain CPCC 205236^T^ are publicly available with the accession numbers OR431689 and GCA_040981255.1, respectively.

#### 5.1.2 *Kocuria sabuli* sp. nov.

*Kocuria sabuli* (sa’bu.li. L. gen. Neut. n. *sabuli*, of sand).

Cells are Gram-stain-positive, aerobic, coccoid (0.8–1.2 μm in diameter), non-motile by peritrichous flagella, non-spore-forming. Colonies are 1.0–2.0 mm in diameter, convex with smooth, round, uniformly edged, opaque and pale orange in color on TSA medium. Growth temperature and pH range for growth are 4–37°C and pH 6.0–11.0, with optima growth at 26–32°C and pH 7.0–9.0. Growth in the presence of up to 10 or 15% (w/v) NaCl. Catalase and oxidase negative. Hydrolysis of starch is positive and cellulose is negative. D-cellobiose, dextrin, gentiobiose, and L-pyroglutamic acid are assimilated. Cells are positive for alkaline phosphatase, esterase (C4), esterase lipase (C8), and *α*-glucosidase and β-glucosidase and negative for acid phosphatase, lipase (C14), cystine arylamidase, α-galactosidase. The polar lipid profiles are diphosphatidylglycerol, phosphatidylglycerol. The major fatty acids (>10%) consist of anteiso-C_15:0_, and C_16:1_ *ω*7c. The predominant quinone is MK-8(H^2^), with minor amount of MK-7(H_2_) and MK-9(H_2_).

The type strain, CPCC 205273^T^ (= KCTC 59062^T^), was isolated from soil from the Badain Jaran Desert, China. The genome size is 4.8 Mbp, and the genomic DNA G + C content is 69.0%. The 16S rRNA gene and whole genome sequence of strain CPCC 205273^T^ are publicly available with the accession numbers OR431691 and GCA_040981185.1, respectively.

#### 5.1.3 *Kocuria cellulosilytica* sp. nov.

*Kocuria cellulosilytica* (cel.lu.lo.si.ly‘ti.ca. N.L. neut. n. *cellulosum*, cellulose; N.L. adj. Lyticus, dissolving; N.L. fem. Adj. cellulosilytica, dissolving cellulose).

Cells are Gram-stain-positive, non-spore-forming, non-motile by flagella, and strictly aerobic. Single cell is coccoid (0.8–1.2 μm in diameter). Colonies are pastel orange. Growth temperature and pH range for growth are 4–42°C and pH 7.0–11.0, with optimum growth at 26–32°C and pH 7.0–9.0. Can tolerate up to 10% (w/v) NaCl. Positive for catalase, hydrolysis of cellulose, gelatin and starch, MR test, cystine arylamidase, esterase lipase (C8), valine arylamidase, and *α*-glucosidase, negative for acid phosphatase (C14), alkaline phosphatase, oxidase, lipase, trypsin, α-chymotrypsin, α-fucosidase, α-galactosidase, *β*-glucosidase, β-glucuronidase, β-galactosidase, α-mannosidase, H_2_S production, siderophore production, and VP test. D-arabitol, D-cellobiose, D-galactose, D-raffinose, D-sorbitol, and L-alanine can be assimilated but D-melibiose, D-salicin, L-arginine. α-D-lactose, and can not. The predominant quinones are MK-7(H_2_), MK-8(H_2_) and MK-9(H_2_). The fatty acid profile consists of the predominant components (> 10%) anteiso-C_15:0_ and C_16:0_.

The type strain, CPCC 205292^T^ (=I19A-01430^T^ = CGMCC 1.60067^T^), was isolated from a sand soil in the Badain Jaran Desert, China. The genome size is 3.9 Mbp, and the genomic DNA G + C content is 72.8%. The 16S rRNA gene and whole genome sequence of strain CPCC 205292^T^ are publicly available with the accession numbers OR431698 and GCA_040981245.1, respectively.

#### 5.1.4 *Kocuria nitroreducens* sp. nov.

*Kocuria nitroreducens* (ni.tro.re.du’cens. N.L. masc. n. *nitras* (gen. nitratis), nitrate; N.L. pref. Nitro-, pertaining to nitrate (in compound words); L. part. Pres. reducens, leading back, bringing back to a more reduced state; N.L. part. Adj. nitroreducens, reducing nitrate).

Cells are aerobic, Gram-stain-positive, coccoid (1.0–1.6 μm in diameter), non-motile and non-spore-forming. Colonies are 1.0–2.0 mm in diameter, convex with smooth, round, uniformly edged, opaque and pale orange in color on TSA medium. Growth temperature and pH range for growth are 4–37°C and pH 6.0–11.0, with optima growth at 26–32°C and pH 7.0–9.0. Cannot tolerate >10% (w/v) NaCl. Catalase-positive and oxidase-negative. Positive for nitrate reduction, gelatin hydrolyzation and starch hydrolysis; negative for cellulose hydrolyzation and H_2_S production. D-arabitol, D-cellobiose, D-salicin, D-sorbitol, L-alanine, gentiobiose, and inosine were assimilated. Activities of alkaline phosphatase, cystine arylamidase, esterase (C4), esterase lipase (C8), leucine arylamidase, naphthol-AS-B1-phosphohydrolase, valine arylamidase and *β*-glucosidase are detected; the following characteristics are absent: acid phosphatase, lipase (C14), N-acetyl-β-glucosaminidase, trypsin, *α*-chymotrypsin, α-fucosidase, α-galactosidase, α-glucosidase, β-glucuronidase, *β*-galactosidase, and α-mannosidase. Diphosphatidylglycerol, phosphatidylglycerol and are detected in the polar lipids extraction. The predominant quinone is MK-8(H_2_), with minor of MK-7(H_2_) and MK-9(H_2_). The fatty acid profile consists of the predominant components (> 10%) anteiso-C_15:0_ and iso-C_15:0_.

The type strain, CPCC 205315^T^ (=KCTC 59068^T^), was isolated from soil in Gurbantunggut Desert, China. The genome size is 4.2 Mbp, and the genomic DNA G + C content is 69.2%. The 16S rRNA gene and whole genome sequence of strain CPCC 205315^T^ are publicly available with the accession numbers OR431660 and GCA_042879195.1, respectively.

#### 5.1.5 *Kocuria oxytropis* sp. nov.

*Kocuria oxytropis* (o.xy.tro’pis. N.L. gen. n. *oxytropis*, of *Oxytropis*, a plant genus, referring to the isolation of the type strain from Oxytropis falcata).

Cells are aerobic, Gram-stain-positive, non-motile, non-spore-forming, coccoid (0.5–0.8 μm in diameter). Colonies are 1.2–2.2 mm in diameter, convex with smooth, round, uniformly edged, opaque and pale orange in color on TSA medium. Growth temperature and pH range for growth are 4–42°C and pH 6.0–11.0, with optima growth at 26–32°C and pH 7.0–9.0. Can tolerate up to 15% (w/v) NaCl. Catalase-positive and oxidase-negative. Positive for cellulose hydrolyzation, gelatin hydrolyzation, nitrate reduction, starch hydrolysis; negative for H_2_S production. Assimilation of D-Cellobiose, Dextrin, D-Salicin, D-Serine, D-Sorbitol, inosine, L-Alanine, and L-Arginine. Activities of alkaline phosphatase, esterase lipase (C8), valine arylamidase, α-glucosidase, and *β*-glucosidase are detected. The following activities are not detected: acid phosphatase, α-galactosidase, β-galactosidase. Diphosphatidylglycerol and phosphatidylglycerol are detected in the polar lipid extraction. The predominant quinone is MK-8(H_2_), with minor of MK-7(H_2_) and MK-9(H_2_). The fatty acid profile (> 10%) consists of the anteiso-C_15:0_ and iso-C_15:0_.

The type strain, CPCC 205268^T^ (= KCTC 59061^T^), was isolated from the rhizosphere soil of *Oxytropis falcata*. The genome size is 4.0 Mbp, and the genomic DNA G + C content is 72.3%. The 16S rRNA gene and whole genome sequence of strain CPCC 206268^T^ are publicly available with the accession numbers OR431690 and GCF_040981195.1, respectively.

### 5.2 Emended species descriptions

#### 5.2.1 Emended description of *Kocuria rosea* (Flügge 1886) (Stackebrandt et al., 1995)

Heterotypic synonym: *Kocuria polaris* (Reddy et al., 2003).

The description is based on the data reported in the (Stackebrandt et al., 1995; Reddy et al., 2003) and this study. Cells are coccoid or spherical (1·0–1·5 μm in diameter), occurring in pairs, tetrads or clusters. Non-motile, Gram-positive, and aerobic. Colonies appear smooth, round, uniformly edged, translucent, mucoid, circular, slightly convex, orange, pink, or red. Grows between 4 and 52°C, with optimum growth at 26–32°C. Grows occur at pH 7–12 and tolerate up to 10% NaCl. Catalase, cystine arylamidase, and lipase-positive. Negative for acid phosphatase, alkaline phosphatase, L-arginine decarboxylase, L-arginine dihydrolase, L-lysine decarboxylase, and *β*-galactosidase. Utilizes acetate, D-cellobiose, D-fructose, D-galactose, D-glucose, D-mannose, D-xylose, glycerol, L-glutamine, L-glycine, L-serine, L-threonine, rhamnose, and sorbitol as sole carbon sources, but can not utilize citrate, D-cysteine, D-ribose, D-sorbose, D-trehalose, dextran, dulcitol, L-histidine, L-isoleucine, L-lysine, L-methionine, L-proline, L-tryptophan, L-tyrosine, L-valine, lactic acid, melezitose, meso-erythritol, succinic acid, sucrose, thioglycollate, and β-hydroxybutyric acid as sole carbon sources. The major fatty acids are *anteiso*-C_15:0_, iso-C_15:0_. The predominant menaquinones are MK-7(H_2_) and MK-8(H_2_). The G + C content of the type strain genome is 72.75 L% and the approximate genome size is 3.88 Mbp. The accession numbers of the 16S rRNA gene and assembly genome sequence are X87756 and GCF_006717035.1, respectively.

The type strain is ATCC 186^T^ (CCM 679^T^ = CCUG 4312^T^ = CIP 71.15^T^ = DSM 20447^T^ = IEGM 394^T^ = IFO 3768^T^ = JCM 11614^T^ = LMG 14224^T^ = NBRC 3768^T^ = NCTC 7523^T^ = NRRL B-2977^T^ = VKM B-1823^T^ = CMS 76or^T^ = MTCC 3702^T^ = DSM 14382^T^).

#### 5.2.2 Emended description of *Kocuria marina*

Heterotypic synonym: *Kocuria indica* (Dastager et al., 2014).

The description is based on the data reported in the previous studies (Kim et al., 2004; Dastager et al., 2014). Cells are Gram-positive, aerobic, non-motile, and coccoid. Catalase-positive, oxidase-negative. Growth occurs in the presence of up to 15% NaCl, although its presence is not required for growth. The temperature range for growth is 4–45°C and optimum growth is observed at 28–30°C. Cellobiose, cupric acid, D-fructose, D-galactose, D-glucose, D-mannitol, D-mannose, D-ribose, inositol, L-rhamnose, lactose, melezitose, melibiose, phenylacetic acid, salicin, sucrose glycogen, trehalose, trisodium citrate, and turanose can be used as sole carbon sources for energy and growth, but not 2-ketogluconate, 5-ketogluconate, aesculin, amygdalin, arabitol, arbutin, D-adonitol, D-arabinose, D-lyxose, D-sorbitol, D-tagatose, dulcitol, erythritol, fucose, gentiobiose, glycerol, inulin, L-arabinose, L-sorbose, lactose, maltose, methyl *α*-D-gluocopyranoside, methyl α-D-mannopyranoside, methyl β-D-xylopyranoside, N-acetylglucosamine, potassium gluconate, raffinose, sucrose, xylitol, xylose. Positive for hydrolysis of starch. The major fatty acid (>10%) contains *anteiso*-C_15:0_, *iso*-C_16:0_ and *anteiso*-C_17:0_. The G + C content of the type strain genome is 68.82% and the approximate genome size is 2.79 Mbp. The accession numbers of the 16S rRNA gene and assembly genome sequence are AY211385 and GCF_014652975.1, respectively.

The type strain is CCUG 51442^T^ (DSM 16420^T^ = JCM 13363^T^ = KCTC 9943^T^ = KMM 3905^T^ = NIO-1021^T^ = NCIM 5455^T^ = DSM 25126^T^ = CCTCC AA 209050^T^).

## Data Availability

The original contributions presented in the study are included in the article/Supplementary material, further inquiries can be directed to the corresponding author.

## Footnotes

^1^ https://lpsn.dsmz.de/genus/kocuria

^2^ https://www.ezbiocloud.net/

^3^ https://www.uniprot.org/

^4^ https://www.ebi.ac.uk/interpro/

^5^ https://antismash.secondarymetabolites.org/

^6^ http://ggdc.dsmz.de/ggdc.php

## Glossary

IAA: Indole-3-acetic acid
MEGA: Molecular evolutionary genetics analysis
NJ: Neighbor-joining
ML: Maximum-likelihood
SOG: Single-copy orthologroups
MLSA: Multilocus sequences analysis
OGRIs: Overall genome relatedness indices
ANI: Average nucleotide identity
dDDH: Digital DNA–DNA hybridization
GGDC: Genome-to-Genome Distance Calculator
BGC: Biosynthetic gene cluster
PYG: Peptone yeast
TSA: Tryptone soya agar
ISP: International Streptomyces Project
YM: Yeast extract sucrose
TSB: Tryptone soya broth
UV: Ultraviolet
API: Analytical profile index
ORF: Open reading frame
COG: Cluster of Orthologous Group
KEGG: Kyoto Encyclopedia of Genes and Genome
phylo-PCA: Phylogenetically informed principal component analysis
TLC: Thin-layer chromatography
HPLC: High performance liquid chromatography
DPG: Diphosphatidylglycerol
PG: Phosphatidylglycerol
NER: Nucleotide excision repair
IAM: Indole-3-acetamide
IPA/IPyA: Indole-3-pyruvic acid
IAN: Indole-3-acetonitrile
TAM: Tryptamine
TSO: L-Trp side-chain oxidase
NAPAA: Non-alpha poly-amino acids like
RiPP-like: Post-translationally modified peptide like
NRPS-like: Non-ribosomal peptide synthetase like
T3PKS: Type III polyketide synthases
DAK: Desert-associated *Kocuria* strains
NDAK: Non-desert associated *Kocuria* strains
Pan-GWAS: Pan-genome-wide association studies
