# Supplementary material for: Genomic insights into Kocuria: taxonomic revision and identification of five IAA-producing extremophiles
Source: Front Microbiol. 2025 May 27;16:1547983. doi: 10.3389/fmicb.2025.1547983 (PMC12149201; doi:10.3389/fmicb.2025.1547983)

**Genomic Insights into *Kocuria*: Taxonomic Revision and Identification of Five IAA-Producing Extremophiles**

Cong-Jian Li^1†^, Zhu-Ming Jiang^1†^, Xiao-Yang Zhi^2^, Hua-Hong Chen^,3^, Li-Yan Yu^1^, Yu-Qin Zhang^1*^

^1^Institute of Medicinal Biotechnology, Chinese Academy of Medical Sciences & Peking Union Medical College, Beijing 100050, P. R. China

^2^Yunnan Institute of Microbiology, Key Laboratory for Conservation and Utilization of Bio-Resource, and Key Laboratory for Microbial Resources of the Ministry of Education, School of Life Sciences, Yunnan University, Kunming, 650091, P. R. China

^3^Yunnan Key Laboratory of Fermented Vegetables, Honghe, 661100, P. R. China

^†^These authors have contributed equally to this work and share the first authorship

**^*^**Author for correspondence:

Yu-Qin Zhang

Tel: +86-10-83167110

Fax: +86-10-83167110

E-Mail: [yzhang@imb.pumc.edu.cn](mailto:yzhang@imb.pumc.edu.cn)

**Supplementary tables:**

All supplementary tables are organized in a Microsoft Excel file named supplementary_tables.xlsx.

**Supplementary Table S1**. The genome information of newly isolated *Kocuria* strains and their close relatives.

**Supplementary Table S2**. The genome information on the 143 *Kocuria* strains from diverse habitats.

**Supplementary Table S3**. Fatty acids profile of eight newly isolated strains and two reference type strains.

**Supplementary Table S4**. The genes related to stress response and IAA production.

**Supplementary Table S5**. Secondary metabolite biosynthesis gene clusters are predicted in the genomes of eight *Kocuria* strains isolated from desert habitats.

**Supplementary Table S6**. The comparison results of COG categories between the core genomes of DAK and NDAK strains.

**Supplementary Table S7**. The comparison results of KEGG pathways between the core genomes of DAK and NDAK strains.

**Supplementary Table S8**. The enriched KOs were associated with the desert habitats by using SCOARY.

**Supplementary figures:**

**Fig. S1. Maximum-likelihood tree showing the phylogenetic relationship between 21 newly isolated strains and 26 type strains of related species among genus *Kocuria* based on 16S rRNA gene sequences.** The bold text represents strains isolated from this study. Bootstrap values are displayed on each branch (1000 replicates), and values greater than 70% are shown. Bar 0.02 represents substitutions per nucleotide position.


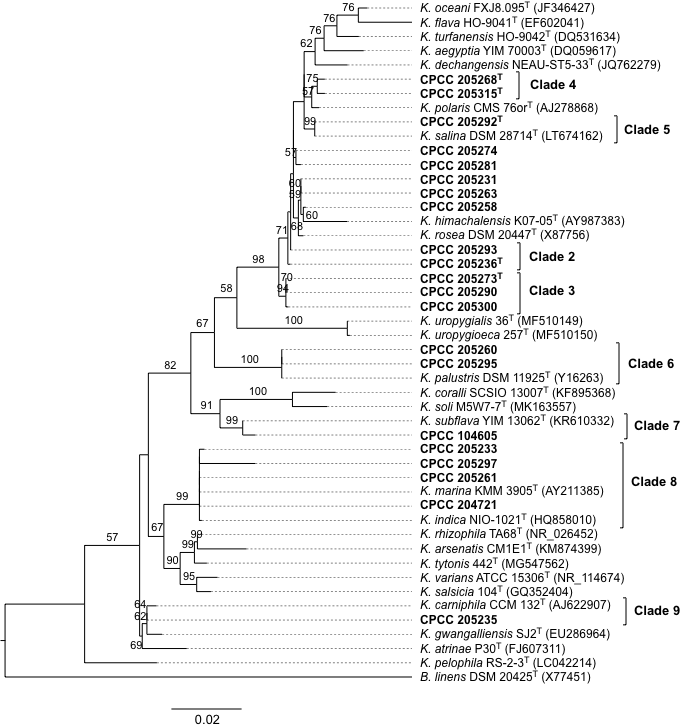


**Fig. S2. A maximum-likelihood tree of 21 newly isolated strains and 26 type strains of related species within the genus *Kocuria* based on 40 universal markers.**

**
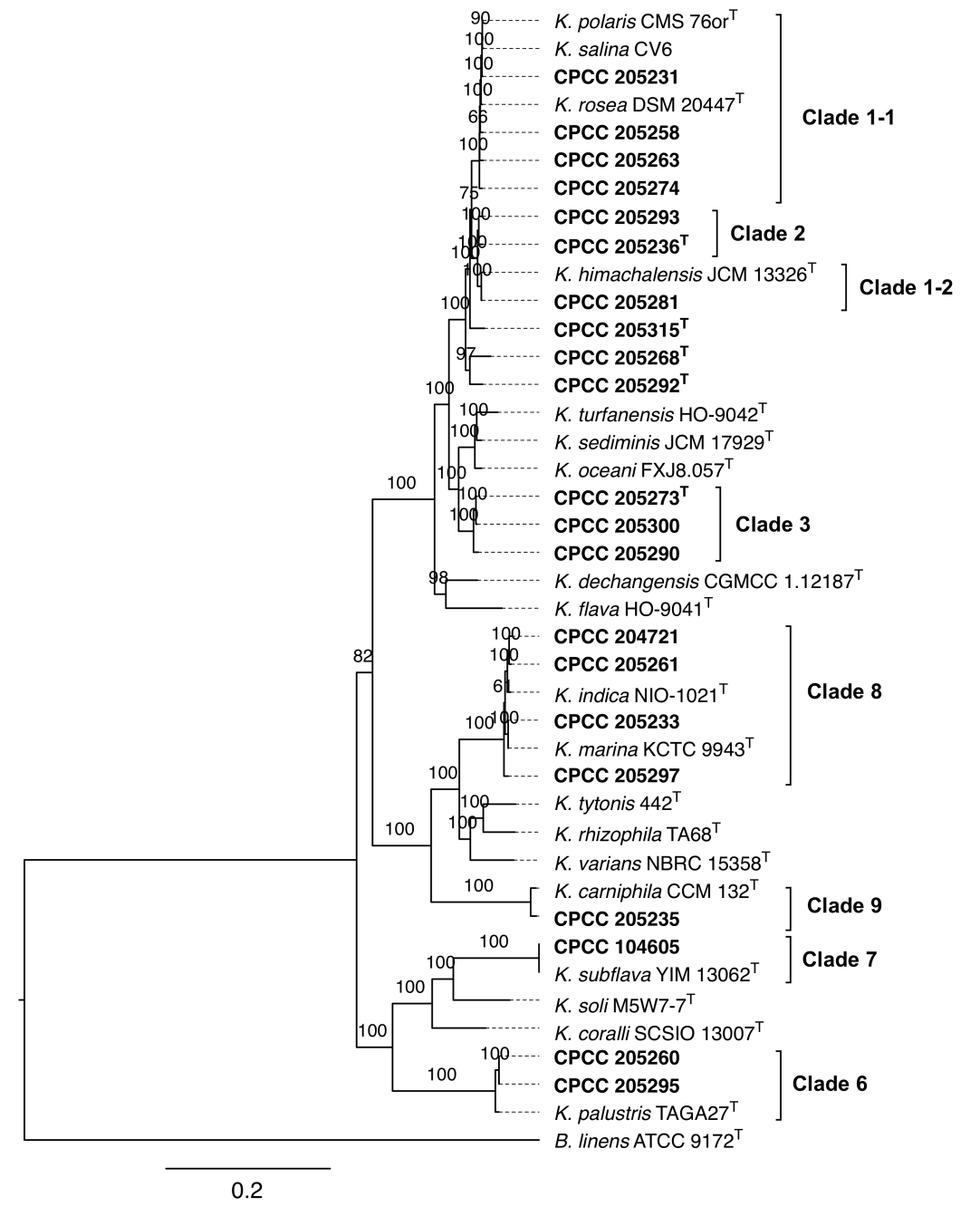
**

**Fig. S3. The ANI values between the 21 newly isolated *Kocuria* strains and strains of their related species.**

**
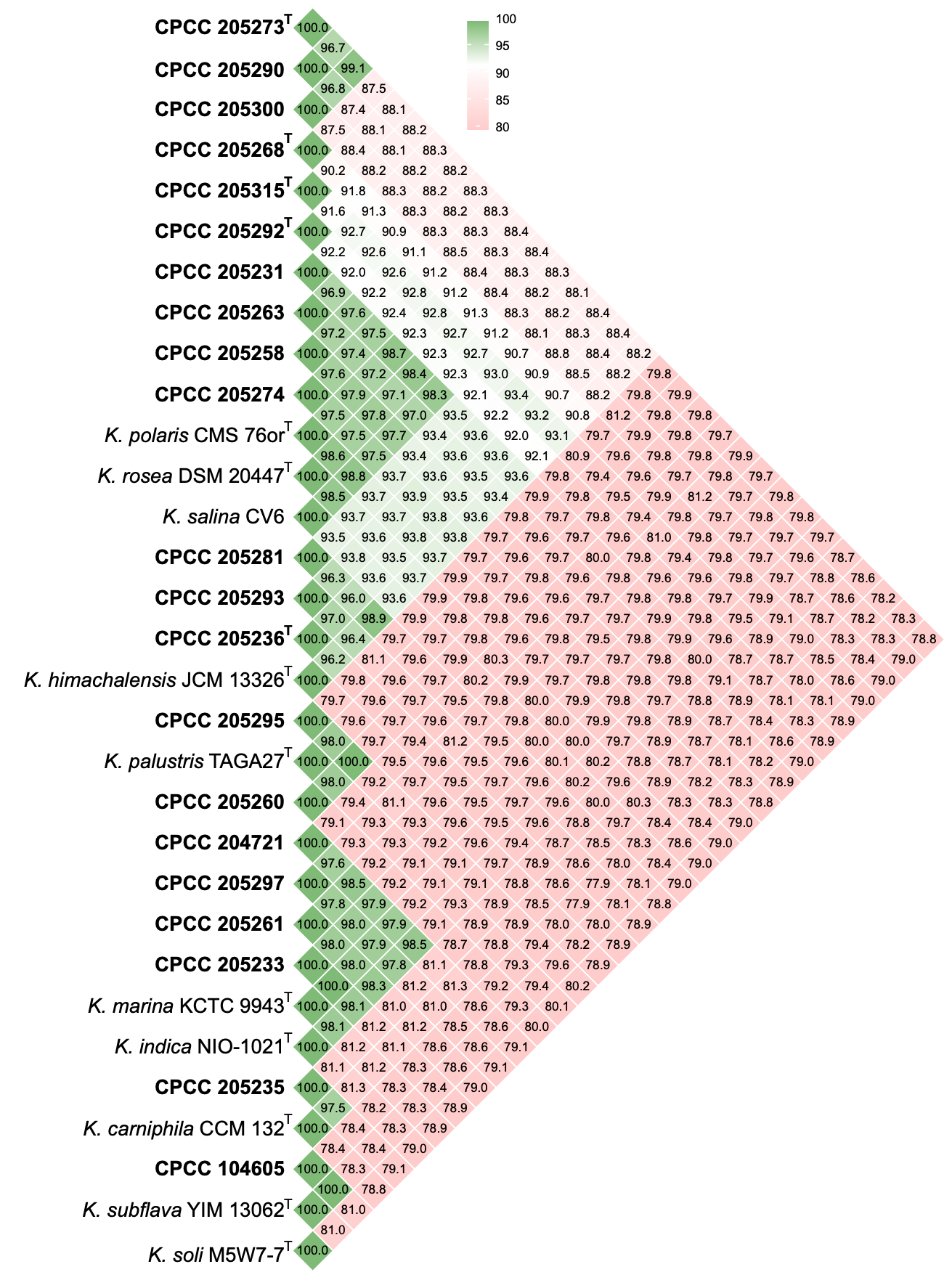
**

**Fig. S4**. **The dDDH values between the 21 newly isolated *Kocuria* strains and strains of their related species.**


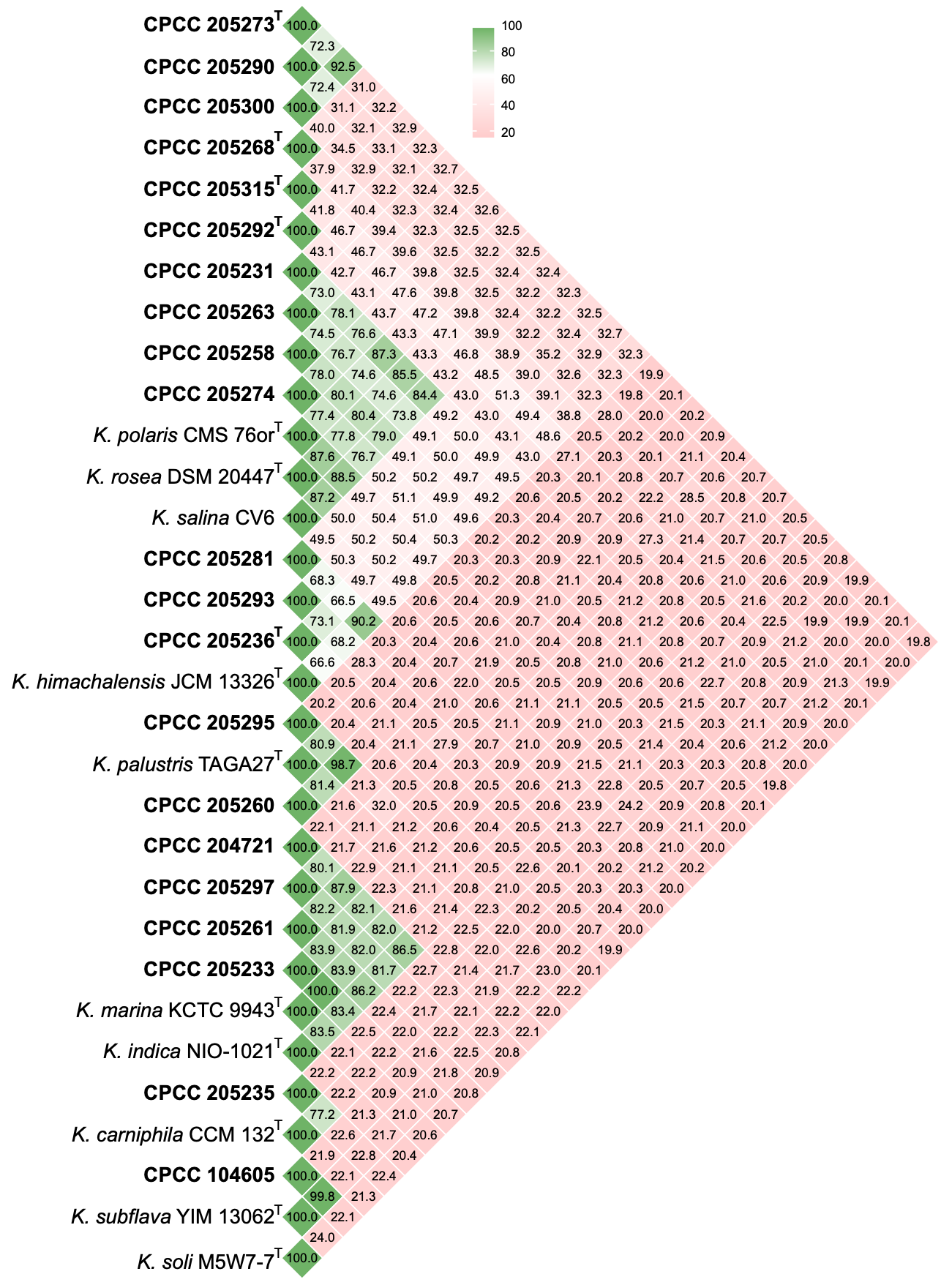


**Fig. S5**. **Two-dimensional thin-layer chromatogram of polar lipids profile of eight newly isolated strains and their closely related type strains.** The spray reagent is 5% molybdatophosphoric acid (1), molybdenum blue (2), and alpha-naphthol (3), respectively. Abbreviations: DPG, diphosphatidylglycerol; PG, phosphatidylglycerol.


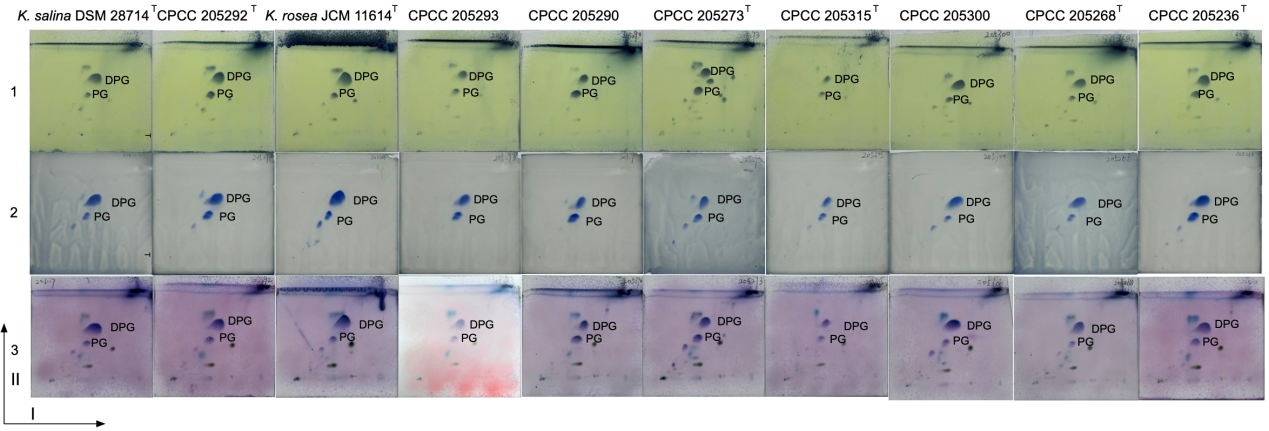


**Fig. S6. Indole acetic acid (IAA) standard curve and absorbance values for fermentation broths of strains CPCC 205236^T^, CPCC 205292^T^, CPCC 205293, CPCC 205290, CPCC 205315^T^, CPCC 205268^T^, *K. rosea* JCM 11614^T^, and *K. salina* DSM 28714^T^ at 530 nm.**


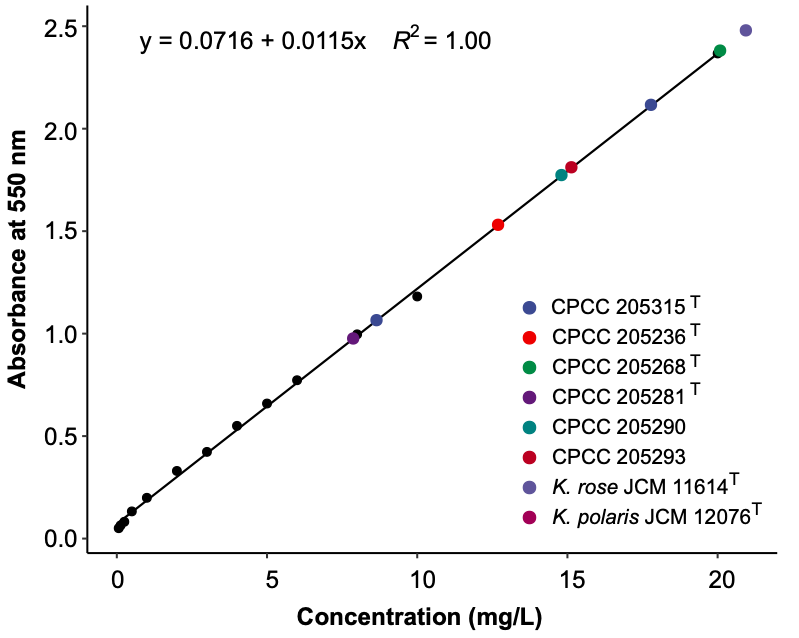

Supplement: Supplementary file 2 [file Data_Sheet_1.docx]
